# ABILITY OF THE PHYSIOLOGICAL PROFILE ASSESSMENT TO CLASSIFY FALLER TYPE: A PROSPECTIVE COHORT STUDY

**DOI:** 10.1093/geroni/igz038.1757

**Published:** 2019-11-08

**Authors:** Deborah A Jehu, Jennifer C Davis, Kristin Velsey, Winnie Cheung, Teresa Liu-Ambrose

**Affiliations:** 1 University of British Columbia, Vancouver, British Columbia, Canada; 2 University of British Columbia - Okanagan Campus, Kelowna, British Columbia, Canada; 3 University of British Columbia, Vancouver, Canada, Canada

## Abstract

Accurately identifying older adults who will experience subsequent falls is important for the provision of secondary fall prevention. The purpose of this study was to determine the accuracy of the Physiological Profile Assessment (PPA) – a valid and reliable fall-risk assessment [1] – in predicting subsequent falls over a 12-month period in older adults who sought for medical attention after an index fall. Seven hundred thirty-seven community-dwelling adults, aged 70 years and older, who were seen at the Vancouver General Hospital Fall Prevention Clinic, completed the PPA at their initial visit. Falls over the subsequent 12 months were tracked prospectively via monthly falls calendars. All individuals received geriatric care at baseline. Binary logistic regressions were performed to determine the accuracy of classifying two prospective faller types: 1) no additional falls; 2) one or more additional fall(s). Baseline PPA, age, and sex were entered as independent variables. During the 12 month observation period, 345 participants had no additional falls (Age:81.3±6.6yrs;Female=251) and 392 fell one or more times (Age:82.3±6.5yrs;Female=230). The classification accuracy was 51.3% for those who had no additional falls and 64.8% for those with one or more additional fall(s) (Overall:58.5%;χ2=29.0;PPA:β=-0.21;Age:β=-0.01;Sex:β=-60). The PPA was not able to accurately differentiate between those who did and did not subsequently fall. Fall-risk assessment sensitivity and specificity should be improved in older adults seeking medical attention following an index fall to inform secondary fall prevention. [1] Lord SR, et al., 2003. Phys Ther.

